# Management of Acute Cholecystitis in High-Risk Patients: Percutaneous Gallbladder Drainage as a Definitive Treatment vs. Emergency Cholecystectomy—Systematic Review and Meta-Analysis

**DOI:** 10.3390/jcm12154903

**Published:** 2023-07-26

**Authors:** Roberto Cirocchi, Lavinia Amato, Serena Ungania, Massimo Buononato, Giovanni Domenico Tebala, Bruno Cirillo, Stefano Avenia, Valerio Cozza, Gianluca Costa, Richard Justin Davies, Paolo Sapienza, Federico Coccolini, Andrea Mingoli, Massimo Chiarugi, Gioia Brachini

**Affiliations:** 1 Department of Medicine and Surgery, S. Maria Hospital, University of Perugia, 05100 Terni, Italy; roberto.cirocchi@unipg.it (R.C.); stefano_avenia@libero.it (S.A.); 2 Department of General and Emergency Surgery, S. Maria della Stella Hospital, 05018 Orvieto, Italy; 3 Department of Digestive and Emergency Surgery, AOSP of Terni, 05100 Terni, Italy; 4 Emergency Department, Policlinico Umberto I, Sapienza University, 00161 Rome, Italy; 5 Department of Emergency Surgery, Fondazione Policlinico Universitario Agostino Gemelli IRCCS, 00168 Rome, Italy; 6 Surgery Center, University Campus Bio-Medico of Rome, 00128 Rome, Italy; 7 Cambridge Colorectal Unit, Addenbrooke’s Hospital, Cambridge University Hospitals NHS Foundation Trust, Cambridge CB2 0QQ, UK; 8 Department of Emergency Surgery, Azienda Ospedaliero, Universitaria of Pisa, 56125 Pisa, Italy

**Keywords:** acute cholecystitis, severe cholecystitis, cholecystectomy, laparoscopic cholecystectomy, open cholecystectomy, cholecystostomy, percutaneous cholecystectomy, gallbladder drain, gallbladder tube, transhepatic gallbladder drain, transhepatic gallbladder tube, cholecystostomy tube

## Abstract

Background: This systematic review aims to investigate whether percutaneous transhepatic gallbladder biliary drainage (PTGBD) is superior to emergency cholecystectomy (EC) as a definitive treatment in high-risk patients with acute cholecystitis (AC). Material and Methods: A systematic literature search was performed until December 2022 using the Scopus, Medline/PubMed and Web of Science databases. Results: Seventeen studies have been included with a total of 783,672 patients (32,634 treated with PTGBD vs. 4663 who underwent laparoscopic cholecystectomy, 343 who had open cholecystectomy and 746,032 who had some form of cholecystectomy, but without laparoscopic or open approach being specified). An analysis of the results shows that PTGBD, despite being less invasive, is not associated with lower morbidity with respect to EC (RR 0.77 95% CI [0.44 to 1.34]; I^2^ = 99%; *p* = 0.36). A lower postoperative mortality was reported in patients who underwent EC (2.37%) with respect to the PTGBD group (13.78%) (RR 4.21; 95% CI [2.69 to 6.58]; *p* < 0.00001); furthermore, the risk of hospital readmission for biliary complications (RR 2.19 95% CI [1.72 to 2.79]; I^2^ = 48%; *p* < 0.00001) and hospital stay (MD 4.29 95% CI [2.40 to 6.19]; *p* < 0.00001) were lower in the EC group. Conclusions: In our systematic review, the majority of studies have very low-quality evidence and more RCTs are needed; furthermore, PTGBD is inferior in the treatment of AC in high-risk patients. The definition of high-risk patients is important in interpreting the results, but the methods of assessment and definitions differ between studies. The results of our systematic review and meta-analysis failed to demonstrate any advantage of using PTGBD over ER as a definitive treatment of AC in critically ill patients, which suggests that EC should be considered as the treatment of choice even in very high-risk patients. Most likely, the inferiority of PTGBD versus early LC for high-risk patients is related to an association of various patient-side factor conditions and the severity of acute cholecystitis.

## 1. Introduction

Acute cholecystitis (AC), typically due to gallstone obstruction of the cystic duct, affects about 200,000 people in the United States each year [1]. For more than a century, open cholecystectomy (OC) was the gold standard [2], while laparoscopic cholecystectomy was introduced in the 1980s [3] and is now the worldwide accepted gold standard [4]. Its success was due to its advantages of less postoperative pain, better cosmetic results and shorter hospital stay with respect to the open approach [5].

In common clinical practice, early laparoscopic cholecystectomy, performed within three days of diagnosis, is the first line of treatment for AC [6,7,8,9,10]. Recently, the WSES (World Society of Emergency Surgery) guidelines suggested performing a laparoscopic cholecystectomy “as soon as possible, within 7 days from hospital admission and within 10 days from the onset of symptoms” [11].

Currently, the choice of treatment is still a dilemma in patients with high risk for severe underlying disease or acutely poor general conditions [12]. In these cases, a percutaneous transhepatic gallbladder drainage (PTGBD) is a minimally invasive technique allowing an immediate drain and decompression of the gallbladder, with a prompt clinical response and few procedure-related complications [13]. In common clinical practice, PTGBD can be preferred as a bridge to surgery over emergency upfront surgery in unfit patients with AC [14,15], but PTGBD as a definitive treatment in extremely high-risk patients is a still matter for debate [16]. Therefore, for each case, a tailored and shared decision-making process is mandatory [17].

However, up until now, few studies managed to compare the results of both approaches, leaving open the discussion of which is the best solution.

This systematic review and meta-analysis of the literature aims to compare two treatment options, PTGBD as a definitive treatment vs. EC, comparing their efficacy and safety in the management of high-risk surgical patients with AC.

## 2. Materials and Methods

The protocol for this systematic review and meta-analysis was registered (CRD42021126286) on the PROSPERO repository (http://www.crd.york.ac.uk/prospero accessed on 15 December 2022). The search of the scientific literature was performed until 15 December 2022, in accordance with the updated PRISMA guidelines for reporting systematic reviews and meta-analyses [18]. The eligibility criteria used for selecting studies were the following: randomized controlled trials (RCTs) and not RCTs in which patients with acute cholecystitis underwent percutaneous gallbladder drainage as a definitive treatment vs. emergency cholecystectomy.

Relevant literature comparing definitive PTGBD vs. EC in AC was searched using the Scopus, Medline/PubMed and Web of Science (Web of Science Category: Surgery) databases. The studies comparing PTGBD vs. conservative medical treatment, those using percutaneous cholecystostomy as a “bridge” to cholecystectomy and those analyzing the outcomes of only one of the two approaches were excluded. In these steps, randomized controlled trials (RCTs), retrospective comparative studies (RCSs), prospective controlled studies (PCSs) and studies from administrative databases (SAD) were selected. All articles were included irrespective of the language. The references of all included studies were checked to identify other relevant papers.

The following keywords were used for the literature search: “acute cholecystitis OR severe cholecystitis OR cholecystectomy OR laparoscopic cholecystectomy OR open cholecystectomy AND cholecystostomy OR percutaneous cholecystostomy OR gall bladder drain OR gallbladder tube OR transhepatic gallbladder drain OR transhepatic gallbladder tube OR cholecystostomy tube.” Two Authors (R.C. and G.B.) independently reviewed the included studies in accordance with the updated PRISMA guidelines for systematic reviews.

To minimize retrieval bias, a manual search was performed through the Google Scholar database. The search for ongoing clinical studies was performed on ClinicalTrials.gov.

The following data were extracted from the studies:

- Authors and year of publication;
- Type of study;
- Countries and facilities involved;
- Timing of treatments;
- Number of patients included;
- Characteristics of patients:
  ○ Age;
  ○ Gender;
  ○ Comorbidities;
  ○ BMI (“body mass index”);
  ○ ASA (“American Society of Anesthesiologists physical status”);
- Diagnostic criteria used for acute cholecystitis;
- Type of percutaneous cholecystostomy:
  ○ Transperitoneal drainage;
  ○ Transhepatic drainage;
- Type of surgical treatment:
  ○ Laparoscopic cholecystectomy;
  ○ Open cholecystectomy.

The primary endpoints were the following:

- Postoperative mortality;
- Emergency surgical treatment;
- Readmission for biliary complications.
- The secondary endpoints were as follows:
- Overall postoperative complications;
- Major postoperative complications;
- Emergency reinterventions;
- Abdominal abscess;
- Length of hospital stay.

The statistical analysis of data was conducted with the Review Manager software (RevMan version 5.4.1) (The Nordic Cochrane Center, The Cochrane Collaboration, Copenhagen, Denmark, 2018).

The results of the first analysis were examined by evaluating the risk ratio for dichotomous variables, while weighted mean differences (WDM) were considered for continuous variables. The statistical model used for the meta-analysis was the random-effects Mantel–Haenszel method, and the results were summarized using forest plots. Heterogeneity of the studies was measured by Cochran’s Q test of heterogeneity and was quantified by Higgins’ index (I^2^) with 95% confidence intervals.

The risk of bias of the included studies was performed by the authors (G.B. and R.C.). A methodological assessment of the included studies was performed with a revised tool for assessing risk of bias in randomized trials (RoB 2) [19] and the Risk of Bias in Non-randomized Studies of Interventions (ROBINS-I) assessment tool [20]. Finally, the GRADE was utilized to perform an analysis of the evidence (GRADEpro GDT. McMaster University and Evidence Prime Inc., Hamilton, ON, Canada, 2021).

## 3. Results

### 3.1. Literature Search

The results of the systematic review of literature are reported in the Supplementary Figure S1. The initial search yielded 2079 potentially relevant articles and 13 articles from additional papers were identified through other sources (grey literature). After removing duplicates and analyzing the remaining titles, abstracts and additional articles, 31 studies remained for full-text analysis. Of these, 14 were excluded because of a lack of data; therefore, only 17 articles were included: 1 RCT [21]; 2 PCS [22,23]; 8 RCS [24,25,26,27,28,29,30,31] and 6 SAD [32,33,34,35,36,37].

**Characteristics of the included studies.** The characteristics of the included studies are summarized in Table 1.

The total number of patients included was 783,672:

- 32,634 treated with percutaneous drainage,
- 4663 underwent laparoscopic cholecystectomy,
- 343 underwent open cholecystectomy,
- 746,032 underwent some form of cholecystectomy, but the authors did not clearly differentiate between the two techniques (laparoscopic or open) [27,31,32,33,35,37].

**Characteristics of included participants.** The characteristics of included cases are reported in Supplementary Table S1.

All studies, excluding Abi Hadair’s, provided information on the gender of the included patients, showing a prevalence of male subjects [24]. All 17 studies reported the age of the patients, but this variable was not comparable. As a matter of fact, the authors used either mean or median age, which are not comparable. However, the patients who underwent percutaneous drainage had an older age with respect to those who had EC. BMI was assessed by a few studies [21,24,25,29]; La Greca et al. reported that the BMI was analyzed, but they did not provide further data in this regard [27]. Surgical risk was assessed in all studies, but different classifications of surgical risk were used. The most common was the ASA scale, but the grade of included patients was very heterogeneous, as reported in Supplementary Table S1. Furthermore, in the study by Melloul et al. the authors preferred SAPS II [28], Loozen et al. used APACHE [21], Latif et al. reported both ASA and APACHE, and others did not specify how they assigned patients into a high-surgical-risk class [30].

**Diagnostic parameters.** The findings of the diagnostic pathways for acute cholecystitis are reported in Supplementary Table S2. Nine of seventeen studies used the diagnostic criteria given by the Tokyo guidelines [15], while SAD used ICD-9 as the database to search for patients with acute cholecystitis. The study by Smith et al. generically indicates that they based the diagnosis on clinical, laboratory and radiological data but provides no further details [31], while the study by Garcés-Albir et al. does not specify how they selected patients [26].

**Type of percutaneous drainage technique performed.** The type of percutaneous gallbladder drainage technique performed is reported in Table 2:

- In seven studies transhepatic access was used;
- In two studies the access was transperitoneal;
- In the study by Loozen et al. [21] both techniques were used;
- Seven studies did not specify the preferred technique.

After the procedure, the PTGBD was removed after a heterogeneous length of time. However, there is no clear standard for this. In the included studies, the time of the PTGBD removal was highly variable, ranging from two to nine weeks. All studies indicated that they removed the catheter after the patients’ clinical condition stabilized. Only the RCT by Loozen et al. [21] suggested that an anterograde cholangiography should be performed before catheter removal to assess duodenal reflux and cystic duct patency.

**Quality assessment of RCTs.** The RCT by Loozen et al. [21] (Supplementary Figure S2) reported a low risk in the evaluation of random sequence generation. The study reports allocation concealment and blinding of outcome assessment and thereby has lower risk of bias.

**Quality assessment of non-RCTs.** According to the ROBINS-I tool, risk-of-bias judgement may be identified as few, moderate, serious or critical (Supplementary Figure S3). Regarding bias due to confounding, five studies were evaluated to have a moderate risk; on the other hand, seven studies have a severe risk and three a critical risk of bias due to confounding. The most common conditions were a higher Charlson Comorbidity Index and the severity of ASA in the PTGBD group. Analyzing the bias in the selection of participants, eight of ten reviews were evaluated as serious risk. The patients’ characteristics were not homogeneous; the patients recruited in the PTGBD group were older than the patients in the EC group. In some studies, a significant difference in BMI was reported. Bias in classifying interventions was severe in seven studies, as they do not report the type of percutaneous access. The length of time associated with percutaneous drainage was not reported in several studies. The bias due to deviation from the intended intervention was serious in all studies. The bias from missing data related to acute cholecystitis was moderate in 6 studies and serious in 10. The bias in measurement of outcomes was moderate in 6 studies and serious in 10.

### 3.2. Analysis of Results: Primary Outcomes

**Figure 1:**
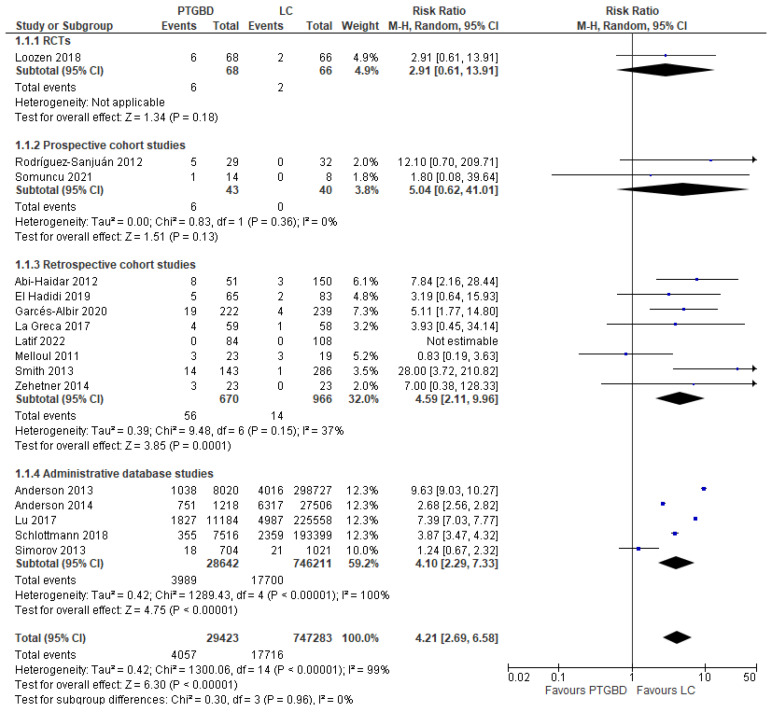
Forest plot of postoperative mortality rates of PTGBD vs. EC [21,22,23,24,25,26,27,28,29,30,31,32,33,34,35,36].

**Postoperative mortality.** This outcome was evaluated in sixteen studies with a total of 776,706 patients. There was a reduction in the incidence of postoperative mortality in the group undergoing cholecystectomy (2.37% (17,716/747,283)) compared to the value found in the group undergoing percutaneous drainage (13.78% (4057/29,423)). The result was statistically significant (RR 4.21; 95% CI [2.69 to 6.58]; *p* < 0.00001). Heterogeneity was extremely high in the overall analysis (I^2^ = 99%) (Figure 1).

The analysis of the different types of studies reported a higher heterogeneity value in the SAD type (I^2^ = 100%) and very low in the other study types: prospective cohort studies (0%) and retrospective cohort studies (37%).

All studies reported lower postoperative mortality in the group undergoing EC:

- RCT: PTGBD group 8.82% (6/68) vs. EC group 3.03% (2/66) (RR 2.91; 95% CI [0.61 to 13.91]; *p* = 0.18).
- Prospective cohort studies: PTGBD group 13.95% (6/43) vs. 0 (0/40) of EC group; this result was not statistically significant (RCTs: RR 2.91 95% CI [0.61 to 13.91]; *p* = 0.18).
- Retrospective cohort: there was an advantage of the EC group (1.45%) (14/966) over the PTGBD group (8.35%) (56/870), and in this case the result was statistically significant (RR 4.59 95% CI [2.11 to 9.96]; *p* = 0.0001).
- Studies from administrative databases: the mortality was 2.37% in the group undergoing cholecystectomy vs. 13.92% in the group undergoing PTGBD, and this difference was statistically significant (RR4.10 95%CI [2.29 to 7.33]; *p* < 0.00001).

Risk of bias was unclear, as studies report general postoperative mortality but not 30-day mortality specifically.

The subgroup analysis of laparoscopic EC was performed in eight studies (1162 patients), and they reported an advantage of laparoscopy with a mortality of 1.98% (12/604) compared to 7.34% (41/558) in the PTGBD group. The result was statistically significant (RR 3.00, 95% CI [1.61 to 5.60]; *p* = 0.0005).

**Figure 2:**
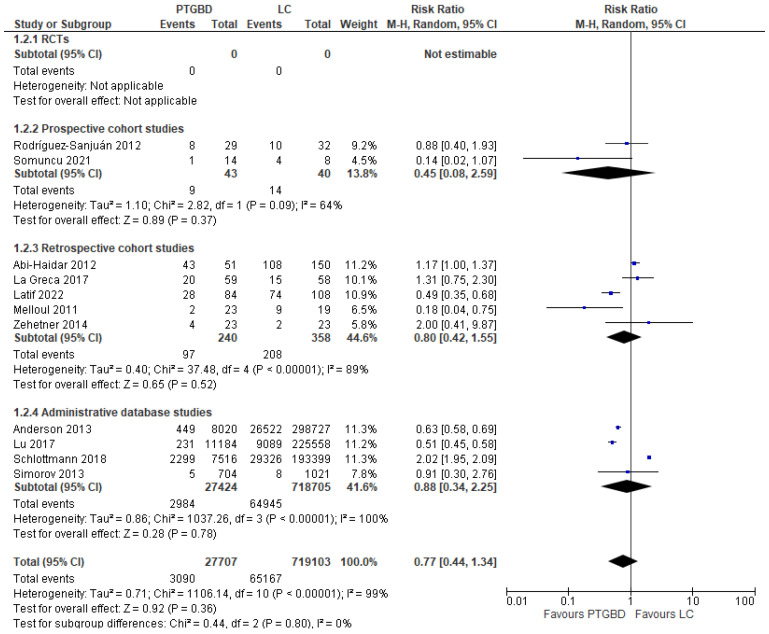
Forest plot of overall postoperative complications between PTGBD and EC [22,23,24,27,28,29,30,32,33,34,35,36].

**Overall postoperative complications.** Eleven studies analyzed this outcome (746,810 patients). The incidence of postoperative complications was similar between the two groups: 11.15% (3090/27,707) in the PTGBD group compared with 9.06% (65,167/719,103) in the EC, but the result did not reach statistical significance (RR 0.77 95% CI [0.44 to 1.34]; I^2^ = 99%; *p* = 0.36). The analysis of different types of studies reported a higher heterogeneity value in SAD (I^2^ = 100%) and retrospective cohort studies (I^2^ = 89%) while it was moderate in prospective cohort studies (64%). Only the analysis of prospective studies reported an advantage of the PTGBD group (20.93%, 9/43) over the EC group (35%, 14/40), but statistical significance was not reached (RR 0.45, 95% CI [0.08 to 2.59]; *p* = 0.37) (Figure 2).

In the analysis of the retrospective group (40.41% in the PTGBD group (97/240) vs. 58.1% in the EC group (208/358)) and SAD (10.88% (2,984/27,424) in the PTGBD group vs. 9.03% (64,945/718,705) in the EC group), the rates were very similar, but statistical significance was reached only in the SAD (RR 0.8 95% CI [0.42 to 1.55]) *p* = 0.52 for the retrospective group, RR 0.88; 95% CI [0.34 to 2.25]) *p* < 0.00001 for the SAD). The risk of bias was found to be unclear; in fact, the authors report only postoperative complications and do not report 30-day mortality specifically.

A subgroup analysis of five LC studies (419 patients) showed a lower rate of complications in the PTGBD group (27.09%) compared to the LC group (48.14%), but the result was not statistically significant (RR 0.59 95% CI [0.27 to 1.29]; *p* = 0.19).

**Figure 3:**
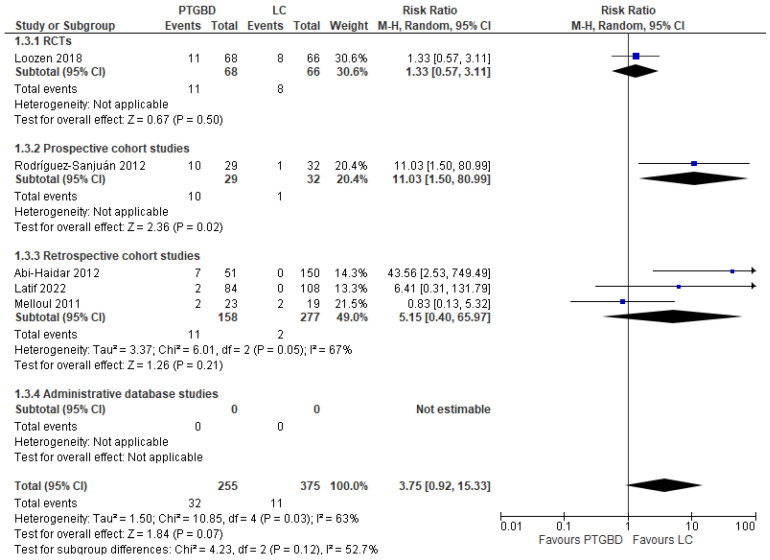
Forest plot of emergency reinterventions between PTGBD and EC (emergency conventional cholecystectomy) [21,22,24,28,30].

**Emergency reinterventions.** This outcome was analyzed in five different studies with a total of 630 patients. The rate of emergency reintervention was lower in the EC group (2.93%) than in the PTGBD group (12.54%), but the result obtained did not reach statistical significance (RR 3.75 95%CI [0.92 to 15.33]; I^2^ = 63%; *p* = 0.07) (Figure 3).

A subgroup analysis of three studies with laparoscopic surgery (368 patients) showed similar results: 5.18% with laparoscopic EC compared with 8.57% with PTGBD. However, the result did not reach statistical significance (RR 1.36 95% CI [0.64 to 2.87]; *p* = 0.42)

**Abdominal Abscess.** Two studies evaluated this outcome (238 patients). The results show a lower incidence of abdominal abscess in the PTGDB group at 0.94% (1/107), compared with 1.53% (2/131) in the EC group (RR 0.70, 95% CI [0.09 to 5.52]; I^2^ = 0%; *p* = 0.73) (Supplementary Figure S4).

### 3.3. Analysis of Results: Secondary Outcomes

**Major postoperative complications.** Four studies reported this endpoint for a total of 485 patients. The incidence of major postoperative complications was higher (30.76%) in the PTGBD group compared with the surgical group (11.15%) but did not reach statistical significance (RR 1.60 CI 95% [0.44 to 5.84]; I^2^ = 86%; *p* = 0.47) (Supplementary Figure S5).

The study by Loozen et al. [21] reported a significant advantage for the EC group with a complication rate of 12.12% compared to 64.7% found in the PTGBD group (RR 5.34 CI 95% [2.72 to 10.46] *p* < 0.00001). While retrospective cohort studies that analyzed this outcome reported an advantage for the PTGBD group, it did not demonstrate statistical significance.

The subgroup analysis of LC studies is comparable to the one of the overall study analyses.

**Length of hospital stay.** Twelve studies have reported this outcome (780,111 patients). Length of hospital stay was statistically lower in the EC group than in the PTGBD group (MD 4.29 95% CI [2.40 to 6.19]; *p* < 0.00001) (Supplementary Figure S6). In the overall analysis, heterogeneity was extremely high (I^2^ = 100%); a subgroup analysis reported a higher heterogeneity value in SAD (I^2^ = 100%), while it was very low in the analysis of prospective cohort studies (0%) and retrospective cohort studies (52%). The analysis of the randomized controlled trial and retrospective cohort studies reported a shorter length of hospital stay in the group that underwent cholecystectomy; however, the result was not statistically significant. In contrast, the analysis of prospective cohort studies and SAD showed a shorter length of hospital stay in the surgical group than in the group undergoing percutaneous drainage, reaching statistical significance.

A subgroup analysis of five LC studies (361 patients) favored the laparoscopic access, and the result reached statistical significance (MD 3.30, 95% CI [1.05 to 5.56]; *p* = 0.004).

**Figure 4:**
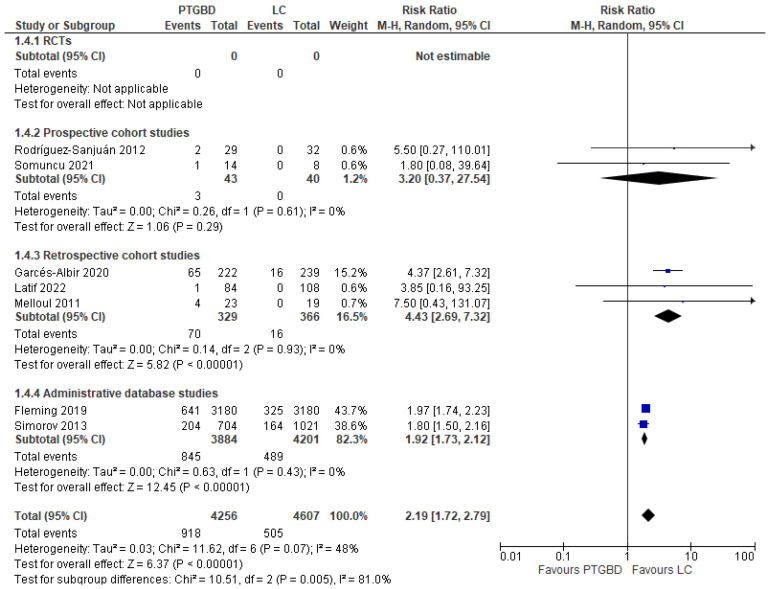
Forest plot of hospital readmission for biliary complications between PTGBD and EC [22,23,26,28,30,34,36].

**Hospital readmission for biliary complications.** Seven studies reported this outcome (8863 patients). The results show a significant increase in the incidence of postoperative complications in the PTGBD group: 21.56% (918/4256) compared with 10.96% (505/4607) in the EC group (RR 2.19 95% CI [1.72 to 2.79]; I^2^ = 48%; *p* < 0.00001) (Figure 4).

In addition, a subgroup analysis of four studies with a total of 717 patients favored laparoscopic access with a readmission rate of 4.27% compared with 20.69% in the PTGBD group, and again, the result reached statistical significance (RR 4.33, 95% CI [2.64 to 7.10] *p* < 0.00001).

### 3.4. Results of GRADE (Grading of Recommendations, Assessment, Development and Evaluations) Analysis

The overall evidence of the four critical outcomes (postoperative 30-day mortality, overall postoperative complications, intra-abdominal abscess and emergency reintervention) was evaluated with GRADE (Table 3) and the quality of evidence was low in all of the analyzed outcomes. For this reason, PTGBD as a definitive treatment should not be used in the treatment of AC due to its lower benefit ratio compared to EC; we suggest that PTGBD as a definitive treatment could be used only in critically ill patients for whom surgery is a very high risk (strength of recommendation “weakly positive”).

## 4. Discussion

In cases of AC in patients with high surgical risk, the Tokyo 2018 guidelines mention PTGBD as an alternative to EC [15]. There are few studies comparing the two different treatment approaches and, to date, only one randomized clinical trial [21]. The studies supporting PTGBD as a definitive treatment are almost all retrospective, dated or based on a limited number of cases [22,23,24,25,26,27,28,29,30,31,32,33,34,35,36,37]; therefore, it can be stated that the current medical literature does not provide solid evidence of the benefits of PTGBD.

This systematic review and meta-analysis were conducted to analyze the advantages and disadvantages of PTGBD as a definitive treatment compared with EC in the management of the critically ill patient with AC. Seventeen studies with a total of 783,672 patients were analyzed.

The study showed that EC is superior to PTGBD in the treatment of high-risk patients with acute cholecystitis. In fact, significant benefits of cholecystectomy were found in terms of lower postoperative mortality (2.37% in the EC group compared with 13.78% in PTGBD) (RR 4.21; 95% CI [2.69 to 6.58]; *p* < 0.00001), decreased incidence of hospital readmission for biliary complications (10.96% in the EC group compared with 21.56% in the PTGBD group) (RR 2.19 95% CI [1.72 to 2.79]; I^2^ = 48%; *p* < 0.00001) and shorter length of hospital stay (MD 3.30, 95% CI [1.05 to 5.56]; *p* = 0.004). The analysis of other endpoints favor EC in the absence of statistically significant values; in fact, a trend towards fewer overall postoperative complications (9.06% vs. 11.15%) (RR 0.77 95% CI [0.44 to 1.34]; I^2^ = 99%; *p* = 0.36), fewer major postoperative complications (11.15 vs. 30.76%) (RR 1.60 CI 95% [0.44 to 5.84]; I^2^ = 86%; *p* = 0.47) and fewer emergency reinterventions (2.93% vs. 12.54%) (RR 3.75 95%CI [0.92 to 15.33]; I^2^ = 63%; *p* = 0.07) was demonstrated in the group undergoing EC compared with the PTGBD group.

The results of this systematic review and meta-analysis are in accordance with the recommendations performed in 2020 from World Society of Emergency Surgery: “*Immediate laparoscopic cholecystectomy is superior to percutaneous transhepatic gallbladder drainage (PTGBD) in high risk patients with ACC. We recommend laparoscopic cholecystectomy as the first-choice treatment in this group of patients*” [11].

The controversies about the use of PTGBD are due to the fact that studies conducted up until now on the subject led to conflicting conclusions. Several observational studies have demonstrated a high short-term success rate of PTGBD in high-risk patients with AC. According to Griniatsos et al. [38], it is possible to perform PTGBD as definite treatment in 100% of the 24 patients included in the study; procedure-related mortality was only 4%, while improvement of the clinical and laboratory picture as well as resolution of sepsis occurred in 90.5% of cases within 72 h of PTGBD. McKay et al. [39] conducted a retrospective study published in 2012 using a larger sample: of the 68 patients included, acute episodes of AC were successfully treated in 58 patients (85%), while 10 patients (15%) died in the hospital. The results of a 10-year experience of PTGBD use in Denmark showed that the procedure-related mortality rate was 4.7 percent, and 234 patients out of the total of 278 were discharged without complications, considering them adequately treated with cholecystostomy [40].

Some authors even suggest PTGBD as a first-line treatment in all high-risk patients with acute cholecystitis [28,32,41,42,43].

However, it is crucial to consider that in support of PTGBD there are mostly retrospective studies with small sample sizes and without control groups (i.e., a group of patients undergoing EC). In addition, many patients who have PTGBD need definitive cholecystectomy at some point after the procedure due to the recurrence of biliary symptoms if the drainage is removed, resulting in morbidity and mortality rates that are generally difficult to monitor and document over time [44,45].

These data suggest that real PTGBD-related mortality may likely be higher than it as reported in the above studies.

Furthermore, when compared to EC, cholecystostomy seems to be related to a higher mortality rate, 10.8% in PTGBD compared to 0.9% in the cholecystectomy group [46], and this difference was statistically significant.

A systematic literature review of 53 studies published in 2009 defines a cholecystostomy as a minimally invasive procedure with a high success rate (85.6%) and low related mortality (0.36%); however, the 30-day mortality is high (15.4%), in contrast to the that related to cholecystectomy (4.5%) [47].

Our systematic review and meta-analysis demonstrate that EC is a safer treatment than PTGBD in critically ill patients.

Another interesting finding is about the rate of hospital readmission for biliary complications, which is significantly higher in the PTGBD than in the EC group (21.56% vs. 10.96%) (RR 2.19 95% CI [1.72 to 2.79]; I^2^ = 48%; *p* < 0.00001). Previous studies showed that performing an upfront cholecystectomy on elderly patients prevents further episodes of gallstone-related disease, reduces readmission rates and is associated with lower overall healthcare costs [48,49,50,51,52].

According to the study by Riall et al. [53], patients who did not undergo cholecystectomy experience gallstone-disease-related hospital readmissions within 2 years more frequently than those who underwent cholecystectomy (38% vs. 4%).

A meta-analysis of 32 studies [54], 9 of them randomized controlled trials, published in 2022, confirmed that the results of the group undergoing cholecystectomy after PTGBD were superior to those of the EC group in terms of postoperative complications (RCTs: RR 0.28, 95% CI 0.14 to 0.56, I^2^ = 63%). The incidence of intra-abdominal abscess, blood loss, conversion to laparotomy, partial cholecystectomy, operative time and wound infection was also lower in the PTGBD group than in the EC group.

If the patients in the present study had undergone elective cholecystectomy after percutaneous drain placement, it is possible that the rate of biliary complications and the risk of hospital readmission would be reduced. On the other hand, one of the advantages of PTGBD is to avoid surgery-related complications, which is why several authors still suggest that patients at high surgical risk should not undergo elective cholecystectomy after percutaneous drainage [38,43,55,56].

According to our experience, only patients particularly at risk for recurrent biliary disease (acute cholecystitis, choledocholithiasis and pancreatitis) should undergo cholecystectomy. Unfortunately, up until now there is no evidence of any clinical, biochemical or radiological predictor of the risk of biliary complications in those patients. Antegrade cholangiography could aid in the selection of patients for elective surgery, but prospective studies on this topic are yet to come.

This meta-analysis, despite the very large number of patients, has limitations that must be acknowledged.

First, some authors, in a large number of patients, did not mention whether an open or laparoscopic surgery was performed.

Second, the selection bias is arising directly from the inclusion of eight retrospective cohort studies. In fact, the higher mortality rate found in patients undergoing cholecystostomy may be due to the fact that PTGBD is usually considered in elderly and severely comorbid patients.

Moreover, there are critical issues related to the use of administrative databases determined mainly by the incompleteness and/or inadequacy of clinical information such as age, gender, BMI or lifestyle information of patients. These confounding factors were not always reported correctly and thus could not be analyzed.

Furthermore, as highlighted in the results, the included studies are heterogenous regarding the criteria used to establish the diagnosis and severity of acute cholecystitis (Tokyo guideline criteria, ICD-9 and clinical, laboratory and radiological data). Surgical risk was also estimated using different classification systems (ASA-PS, APACHE II and SAPS II). These are key factors that contributed to the heterogeneity of the meta-analysis.

A further concern is about the selection of patients to be treated with PC. This approach was reserved for subjects considered to be at a higher operative risk, such as those that are elderly, that are comorbid, or with high-grade cholecystitis; this may account for the greater bias in the data collected. A recent study of Serban et al. reported that diabetes was associated with a higher rate of surgical and systemic postoperative morbidity; differently, stroke and chronic renal insufficiency were associated with a major risk of cardiovascular complications [57].

TG18 recommends that PTGBD followed by Lap-C should be performed first in high-risk patients for emergency surgical treatment. That is, it is recommended to perform PTGBD as a bridge to elective surgery. Successively, some surgeons suggest considering PTGBD a definitive treatment in high-risk patients [15]. However, more recent evidence shows that those guidelines may have overestimated the role of PTGBD and that these patients would obtain a larger benefit from cholecystectomy [21,46]. It must be considered that the only randomized trial published until now on this topic is the CHOCOLATE study by Loozen et al. [21], which showed that laparoscopic cholecystectomy is better than percutaneous drainage in high-risk patients with acute cholecystitis. The aforementioned study demonstrated that LC was associated with lower postoperative mortality (3% vs. 9%), a lower rate of major complications (12% vs. 65%), lower incidence of cholecystitis-related reinterventions (12% vs. 66%), lower recurrence of biliary disease (5% v 53%) and also shorter hospital stay including readmissions (5 days vs. 9 days) [21]. PTGBD may be an acceptable treatment choice when surgery is absolutely contraindicated, i.e., a recent myocardial infarction (MI) (<6 weeks) or acute cerebrovascular accident.

In view of the detected bias of the published studies and the results obtained in the present systematic review, it is clear that further studies with stricter criteria are needed to evaluate clinical, biochemical and/or radiological factors, which are predictive of the failure of the percutaneous drainage in acute cholecystitis. Strong evidence-based guidelines, as reported from Hung et al. [58], are most needed on the real indications for PTGBD, specifying the timing with respect to the onset of symptoms, the route of catheter insertion (transhepatic or transperitoneal) and the length of time the catheter should remain in place. Now, it is also extremely difficult to compare the results of PTGBD from different centers. On the contrary, indications, techniques and follow-ups of PTGBD should be standardized so that it would be possible to select patients who could truly benefit from this treatment and those who, on the contrary, could be candidates for upfront emergency cholecystectomy.

## 5. Conclusions

In this systematic review, the majority of studies have very low-quality evidence and more RCTs are needed; furthermore, the meta-analysis reported that PTGBD is inferior in the treatment of AC in high-risk patients and there is no demonstrated advantage in using PTGBD as a definitive treatment over EC in the management of critically ill patients with acute cholecystitis. Another important limitation of this study is the definition of high-risk patients; in fact, these data are very important in interpreting the results, but the methods of assessment and definitions differ between studies.

The analysis of our results shows that PTGBD, despite being minimally invasive, is burdened with a higher incidence of complications with respect to EC. The lower postoperative mortality, lower risk of hospital readmission for biliary complications and shorter hospital stay times suggest considering upfront cholecystectomy as the treatment of choice, even for patients at high surgical risk.

Most likely, the inferiority of PTGBD versus early LC for high-risk patients is related to an association of various patient-side factor conditions and the severity of acute cholecystitis. Currently, percutaneous cholecystostomy should be reserved only for patients with very poor general conditions on whom surgery is not feasible or appropriate, or as a bridge to elective surgery in patients whose general conditions can be improved after an initial damage-control procedure.

## Figures and Tables

**Table 1:** Characteristics of the included studies (RCS: retrospective cohort study, PCS: prospective cohort study, NR: not reported, LC: laparoscopic cholecystectomy, OC: open cholecystectomy, PTGBD: percutaneous transhepatic gallbladder biliary drainage).

Author and Year of Publication	Nation	Type of Study	N. of Patients Included	Time of Enrolment	PTGBD Group		LC in Control Group		OC		NR
					N. of Patients	Timing	N. of Patients	Timing	N. of Patients	Timing	N. of Patients
Latif et al., 2022 [30]	United Kingdom	RCS	192	2016 to 2018	84	NR	108	After >48 of conservative management	0	NR	0
Somuncu et al., 2021 [23]	Turkey	PCS	22	March 2020 to June 2020	14	NR	8	NR	0	NR	0
Garcés Albir et al., 2020 [26]	Spain–Uruguay	RCS	461	January 2005 to December 2016	222	NR	239	NR	0	NR	0
El Hadidi et al., 2019 [25]	Egypt	RCS	225	February 2014 to September 2017	65	NR	83	NR	77	NR	0
Fleming et al., 2019 [34]	USA	Administrative database studies	6360	2013 to 2014	3180	NR	3180	NR	0	0	0
Loozen et al., 2018 [21]	Netherlands	RCT	134	February 2011 to January 2016	68	Within 24 h after randomization	66	Within 24 h after randomization	0	0	0
Schlottmann et al., 2018 [35]	USA–Argentina	Administrative database studies	200,915	January 2000 to December 2014	7516	NR	NR	NR	NR	NR	193,399
La Greca et al., 2017 [27]	Italy	RCS	646	August 2009 to March 2016	90	NR	NR	NR	NR	NR	556
Lu et al., 2017 [37]	Taiwan	Administrative database studies	236,742	2003 to 2012	11,184	NR	NR	NR	NR	NR	225,558
Anderson et al., 2014 [33]	USA	Administrative database studies	28,724	1995 to 2009	1218	NR	NR	NR	NR	NR	27,506
Zehetner et al., 2014 [29]	USA	RCS	46	January 1999 to October 2010	23	NR Presence of symptoms for 72 h	23	NR Presence of symptoms for 72 h	0	0	0
Anderson et al., 2013 [32]	USA	Administrative database studies	306,747	1998 to 2010	8020	NR	NR	NR	NR	NR	298,727
Simorov et al., 2013 [36]	USA	Administrative database studies	1725	October 2007 to June 2011	704	NR	822	NR	199	NR	0
Smith et al., 2013 [31]	USA	RCS	432	April 1998 to December 2009	143	NR	NR	NR	NR	NR	286
Abi-Haidar et al., 2012 [24]	USA	RCS	201	January 2001 to December 2010	51	NR	110	During 24 h of admission = 32 Later than 24 h after admission = 33 Elective procedure = 45	40	During 24 h of admission = 26 Later than 24 h after admission = 7 Elective procedure = 3 Emergency procedure after discharged = 4	0
Rodrìguez-Sanjuàn et al., 2012 [22]	Spain	PCS	61	January 2005 to December 2010	29	NR	14	First 72 h from AC onset	18	First 72 h from AC onset	0
Melloul et al., 2011 [28]	Switzerland	RCS	42	2001 to 2007	23	12–24 h	10	12–24 h	9	12–24 h	0
Total			783,672		32,634		4663		343		746,032

**Table 2:** Type of percutaneous drainage technique performed.

Transhepatic Route	Transperitoneal Route	Not Reported
Latif et al., 2022 [30]	Loozen et al., 2018 [21]	Fleming et al., 2019 [34]
Somuncu et al., 2021 [23]	La Greca et al., 2017 [27]	Schlottmann et al., 2018 [35]
Garcés Albir et al., 2020 [26]	Smith et al., 2013 [31]	Lu et al., 2017 [37]
El Hadidi et al., 2019 [25]		Anderson et al., 2014 [33]
Loozen et al., 2018 [21]		Zehetner et al., 2014 [29]
Melloul et al., 2011 [28]		Anderson et al., 2013 [32]
Abi-Haidar et al., 2012 [24]		Simorov et al., 2013 [36]
Rodriguez-Sanjuàn et al., 2012 [22]		

**Table 3:** Summary of findings.

PTGBD Compared to EC						
**Patient or population:** Acute cholecystitis **Setting:** Emergency admission **Intervention:** PTGBD (percutaneous transhepatic gallbladder biliary drainage) **Comparison:** EC (emergency cholecystectomy)						
Outcomes	Anticipated absolute effects * (95% CI)		Relative effect (95% CI)	No. of participants (studies)	Certainty of the evidence (GRADE)	Comments
	**Risk with placebo**	**Risk with Postoperative**				
Postoperative 30-day mortality	24 per 1000	**100 per 1000** (64 to 156)	**RR 4.21** (2.69 to 6.58)	776,706 (16 studies)	⨁⨁◯◯ Low	PTGBD could be used in critical-ill-condition patients for whom surgery is a very high risk (recommendation “positive weak”)
Overall postoperative complications	91 per 1000	**70 per 1000** (40 to 121)	**RR 0.77** (0.44 to 1.34)	746,810 (11 studies)	⨁⨁◯◯ Low	PTGBD could be used in critical-ill-condition patients for whom surgery is a very high risk (recommendation “positive weak”)
Intra-abdominal abscess	15 per 1000	**11 per 1000** (1 to 84)	**RR 0.70** (0.09 to 5.52)	238 (2 studies)	⨁⨁◯◯ Low	PTGBD could be used in critical-ill-condition patients for whom surgery is a very high risk (recommendation “positive weak”)
Emergency reintervention	29 per 1000	**110 per 1000** (27 to 450)	**RR 3.75** (0.92 to 15.33)	630 (5 studies)	⨁⨁◯◯ Low	PTGBD could be used in critical-ill-condition patients for whom surgery is a very high risk (recommendation “positive weak”)
* **The risk in the intervention group** (and its 95% confidence interval) is based on the assumed risk in the comparison group and the relative effect of the intervention (and its 95% CI). **CI:** confidence interval; **RR:** risk ratio						
**GRADE Working Group grades of evidence** **High certainty:** we are very confident that the true effect lies close to that of the estimate of the effect. **Moderate certainty:** we are moderately confident in the effect estimate: the true effect is likely to be close to the estimate of the effect, but there is a possibility that it is substantially different. **Low certainty:** our confidence in the effect estimate is limited: the true effect may be substantially different from the estimate of the effect. **Very low certainty:** we have very little confidence in the effect estimate: the true effect is likely to be substantially different from the estimate of effect.						

## Data Availability

Not applicable.

## Supplementary Materials

The following supporting information can be downloaded at: https://www.mdpi.com/article/10.3390/jcm12154903/s1, Figure S1. Prisma flow chart of literature search; Figure S2. Risk-of-bias summary: review authors’ judgments about each risk-of-bias item for included studies; Figure S3. Risk-of-bias domains; Figure S4. Forest plot of abdominal abscess in PTGBD vs. EC; Figure S5. Forest plot of major postoperative complications between PTGBD vs. EC; Figure S6. Forest plot of length of hospital stay between PTGBD vs. EC; Table S1. Characteristics of included participants; Table S2. Diagnostic parameters in included studies.
