# Supplementary material for: Management of Acute Cholecystitis in High-Risk Patients: Percutaneous Gallbladder Drainage as a Definitive Treatment vs. Emergency Cholecystectomy—Systematic Review and Meta-Analysis
Source: J Clin Med. 2023 Jul 26;12(15):4903. doi: 10.3390/jcm12154903 (PMC10419867; doi:10.3390/jcm12154903)

Supplementary Figure S2. Risk-of-bias summary: review authors' judgments about each risk-of-bias item for included studies.

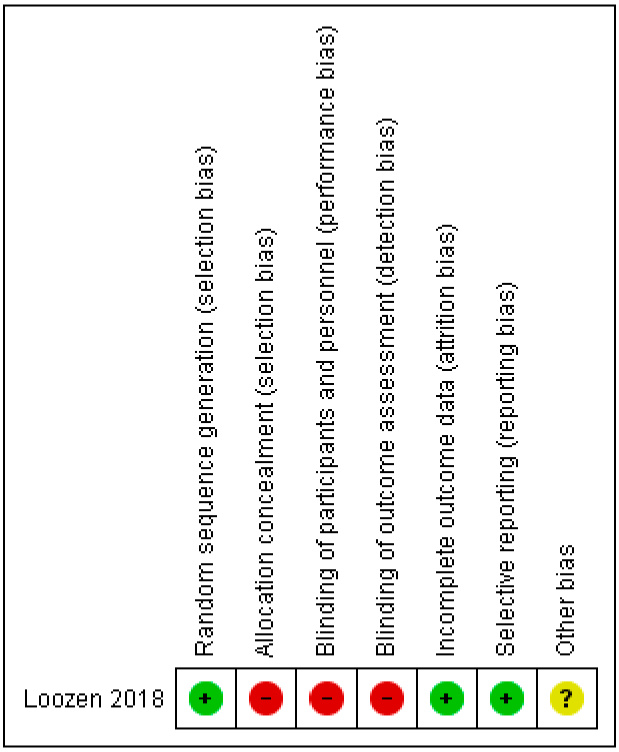

Supplement: Supplementary file 1 [file jcm-12-04903-s001.zip › SDC Fig 2.pdf]
