# Supplementary material for: Management of Acute Cholecystitis in High-Risk Patients: Percutaneous Gallbladder Drainage as a Definitive Treatment vs. Emergency Cholecystectomy—Systematic Review and Meta-Analysis
Source: J Clin Med. 2023 Jul 26;12(15):4903. doi: 10.3390/jcm12154903 (PMC10419867; doi:10.3390/jcm12154903)
Supplement: Supplementary file 1 [file jcm-12-04903-s001.zip › SDC Tab 1.pdf]

Supplementary Table S1. Characteristics of included participants.

Author - year	Type of treatment	N. of patients	Age	Sex (M/F)	BMI	ASA
Latif et al. 2022	PTGBD group	84	Median age:74	51.19% /48.81 (43/41)	NR	I=35 II=43 III=6 IV=0
	LC in control group	108	Median age: 46	28.7%/71,3 (31/77)	NR	I=90 II=18 III/IV=0
	OC in control group	0	0	0	0	0
	NR	0	0	0	0	0
Somuncu et al. 2021	PTGBD group	14	Median age: 68	7/7	NR	IV or more
	LC in control group	8	Median age: 44	4/4	NR	NR
	OC in control group	0	0	0	0	0
	NR	0	0	0	0	0

Garcés Albir et al. 2020	PTGBD group	222	Mean Age (SD): 78.63 ±10.91	115/107	NR	I/II = 54 III/IV = 165
	LC in control group	239	Mean Age (SD): 74.06±9.36	138/101	NR	I/II = 127 III/IV = 112
	OC in control group	0	0	0	0	0
	NR	0	0	0	0	0
El Hadidi et al. 2019	PTGBD group	65	Mean Age (SD): 65.8±1.73.6	22/43	26±0.4	III and IV
	LC in control group	83	Mean Age (SD): 75±9.7	30/53	24.8±0.6	I and II
	OC in control group	77	Mean Age (SD): 66.6±5.4	27/50	26.4±0.7	I and II
	NR	0	0	0	0	0
	PTGBD group	3180	Division in subgruop	1726/1454	NR	NR

Fleming et al. 2019	LC in control group	3180	Division in subgruop	1750/1430	NR	NR
	OC in control group	0	0	0	0	0
	NR	0	0	0	0	0
Loozen et al. 2018	PTGBD group	68	Mean Age (SD): 74.9 (8.6)	44/24	29.0 (5.5)	I = 4<br>II = 37<br>III = 24<br>IV = 3
	LC in control group	66	Mean Age (SD): 71.4 (10.6)	41/25	28.7 (5.3)	I = 10<br>II = 33<br>III = 23<br>IV = 0
	OC in control group	0	0	0	0	0
	NR	0	0	0	0	0
Schlottmann et al. 2018	PTGBD group	7516	Median Age = 80	4033/3483	NR	NR
	LC in control group	0	0	0	0	0

	OC in control group	0	0	0	0	0
	NR	193399	Median Age = 75	89759/103557<br>83 not specified.	NR	NR
La Greca et al. 2017	PTGBD group	90	Mean Age (SD): 78.3 ± 11.4	56/34	Used, but not specified	I =3<br>II =28<br>III =59
	LC in control group	0	0	0	0	0
	OC in control group	0	0	0	0	0
	NR	556	Mean Age (SD): 55.5 ± 17.6	268/288	Used, but not specified	I =276<br>II =222<br>III = 58
Lu et al. 2017	PTGBD group	11184	Divided in subgroup	59,14% / 40.86% (86614/4570)	NR	NR

	LC in control group	NR	0	0	0	0
	OC in control group	NR	0	0	0	0
	NR	225558	Divided in subgroup	49,22% / 50,78% (111020/114538)	NR	NR
Anderson et al. 2014	PTGBD group	1218	Mean Age (SD): 71.2 ± 15.0	666/552	NR	NR
	LC in control group	0	0	0	0	0
	OC in control group	0	0	0	0	0
	NR	27506	Mean Age (SD): 57.2 ± 19.1	13610/13896	NR	NR

Zehetner et al. 2014	PTGBD group	23	Mean Age (SD): 57.3 (±14.7)	7/16	28.3 (±5.8)	NR
	LC in control group	23	Mean Age (SD): 49.8 (±13.1)	11/12	28.2 (±8.8)	NR
	OC in control group	0	0	0	0	0
	NR	0	0	0	0	0
Anderson et al. 2013	PTGBD group	8020	Mean Age: 71,87	4489/3531	NR	NR
	LC in control group	0	0	0	0	0
	OC in control group	0	0	0	0	0
	NR	298727	Mean age: 54,81	122324/176403	NR	NR
Simorov et al. 2013	PTGBD group	704	Divided in subgroup	39.1%/60.9% (428,736/275,264)	NR	NR

	LC in control group	822	Divided in subgroup	58,4%/41,6% (480,048/341,952)	NR	NR
	OC in control group	199	Divided in subgroup	65,3%/34,7% (129,95/69,05)	NR	NR
	NR	0	0	0	0	0
Smith et al. 2013	PTGBD group	143	Mean age (SD) 72.0 (±13.5)	65%/35% (50/93)	NR	I=1 II=25 III=74 IV=43
	LC in control group	0	0	0	0	0
	OC in control group	0	0	0	0	0
	NR	286	Mean age (SD) 56.6 (±18.8)	40%/60% 115/171	NR	I=30 II=164 III=80 IV=12
	PTGBD group	51	70.4[13.9]	NR	28.0 (5.4)	III-IV = 45

Abi-Haidar et al. 2012	LC in control group	110				
	OC in control group	40				
	NR		65.0[13.3]	NR	29.7 (5.6)	III-IV = 103
Rodrìguez-Sanjuàn et al. 2012	PTGBD group	29	Mean Age: 81.8	18/11	NR	II = 2 III = 1 IV = 12
	LC in control group	14				
	OC in control group	18				
	NR		Mean Age: 83.6	19/13	NR	II=0 III = 29 IV = 3
	PTGBD group	23	Median age = 65	18/5	NR	Used SAPS

						SAPS II > 15 = high risk
Melloul et al. 2011	LC in control group	10				
	OC in control group	9				
	NR		Median age = 63	16/3	NR	NR
