# Supplementary material for: Management of Acute Cholecystitis in High-Risk Patients: Percutaneous Gallbladder Drainage as a Definitive Treatment vs. Emergency Cholecystectomy—Systematic Review and Meta-Analysis
Source: J Clin Med. 2023 Jul 26;12(15):4903. doi: 10.3390/jcm12154903 (PMC10419867; doi:10.3390/jcm12154903)
Supplement: Supplementary file 1 [file jcm-12-04903-s001.zip › SDC Tab 2.pdf]

Supplementary Table S2. Diagnostic parameters in included studies

Author – year		Physical examination	Laboratory findings		Ultrasonographic abnormalities		
		**Tenderness**	**Elevated leukocyte**	**C-reactive protein levels**	**Gallstones, sludge**	**Wall thickening ≥ 3 mm, pericholecystic fluid**	**Murphy's sign**
Latif et al 2022	**PTGBD group**	Yes	Yes	Yes	Yes	Yes	Yes
	**LC in control group**	Yes	Yes	Yes	Yes	Yes	Yes
	**OC in control group**	Yes	Yes	Yes	Yes	Yes	Yes
Somuncu et al 2021	**PTGBD group**	NR	NR	NR	NR	Yes	NR
	**LC in control group**	NR	NR	NR	NR	Yes	NR

Author – year		Physical examination	Laboratory findings		Ultrasonographic abnormalities		
		Tenderness	Elevated leukocyte	C-reactive protein levels	Gallstones, sludge	Wall thickening ≥ 3 mm, pericholecystic fluid	Murphy's sign
	**OC in control group**	Yes	Yes	Yes	NR	Yes	NR
Garcés Albir et al 2020	**PTGBD group**	NR	NR	NR	NR	NR	NR
	**LC in control group**	NR	NR	NR	NR	NR	NR
	**OC in control group**	NR	NR	NR	NR	NR	NR
El Hadidi et al 2019	**PTGBD group**	Yes	Yes	Yes	Yes	Yes	Yes
	**LC in control group**	Yes	Yes	Yes	Yes	Yes	Yes

Author – year		Physical examination	Laboratory findings		Ultrasonographic abnormalities		
		**Tenderness**	**Elevated leukocyte**	**C-reactive protein levels**	**Gallstones, sludge**	**Wall thickening ≥ 3 mm, pericholecystic fluid**	**Murphy's sign**
	**OC in control group**	Yes	Yes	Yes	Yes	Yes	Yes
Fleming et al 2019	**PTGBD group**	NR	NR	NR	NR	NR	NR
	**LC in control group**	NR	NR	NR	NR	NR	NR
	**OC in control group**	NR	NR	NR	NR	NR	NR
Loozen et al 2018	**PTGBD group**	Yes	Yes	Yes	Yes	Yes	Yes
	**LC in control group**	Yes	Yes	Yes	Yes	Yes	Yes

Author – year		Physical examination	Laboratory findings		Ultrasonographic abnormalities		
		Tenderness	Elevated leukocyte	C-reactive protein levels	Gallstones, sludge	Wall thickening ≥ 3 mm, pericholecystic fluid	Murphy's sign
Schlottmann et al 2018	PTGBD group	NR	NR	NR	NR	NR	NR
	LC in control group	NR	NR	NR	NR	NR	NR
	OC in control group	NR	NR	NR	NR	NR	NR
La Greca et al 2017	PTGBD group	Yes	Yes	Yes	Yes	Yes	Yes
	LC in control group	Yes	Yes	Yes	Yes	Yes	Yes
	OC in control group	Yes	Yes	Yes	Yes	Yes	Yes

Author – year		Physical examination	Laboratory findings		Ultrasonographic abnormalities		
		Tenderness	Elevated leukocyte	C-reactive protein levels	Gallstones, sludge	Wall thickening ≥ 3 mm, pericholecystic fluid	Murphy's sign
Lu et al. 2017	**PTGBD group**	NR	NR	NR	NR	NR	NR
	**LC in control group**	NR	NR	NR	NR	NR	NR
	**OC in control group**	NR	NR	NR	NR	NR	NR
Anderson et al 2014	**PTGBD group**	NR	NR	NR	NR	NR	NR
	**LC in control group**	NR	NR	NR	NR	NR	NR
	**OC in control group**	NR	NR	NR	NR	NR	NR

Author – year		Physical examination	Laboratory findings		Ultrasonographic abnormalities		
		Tenderness	Elevated leukocyte	C-reactive protein levels	Gallstones, sludge	Wall thickening ≥ 3 mm, pericholecystic fluid	Murphy's sign
Zehetner et al 2014	**PTGBD group**	Yes	Yes	NR	Yes	Yes	NR
	**LC in control group**	Yes	Yes	NR	Yes	Yes	NR
	**OC in control group**	Yes	Yes	NR	Yes	Yes	NR
Anderson et al 2013	**PTGBD group**	NR	NR	NR	NR	NR	NR
	**LC in control group**	NR	NR	NR	NR	NR	NR
	**OC in control group**	NR	NR	NR	NR	NR	NR

Author – year		Physical examination	Laboratory findings		Ultrasonographic abnormalities		
		Tenderness	Elevated leukocyte	C-reactive protein levels	Gallstones, sludge	Wall thickening ≥ 3 mm, pericholecystic fluid	Murphy's sign
Simorov et al 2013	**PTGBD group**	NR	NR	NR	NR	NR	NR
	**LC in control group**	NR	NR	NR	NR	NR	NR
	**OC in control group**	NR	NR	NR	NR	NR	NR
Smith	**PTGBD group**	NR	NR	NR	NR	NR	NR
	**LC in control group**	NR	NR	NR	NR	NR	NR
	**LC in control group**	NR	NR	NR	NR	NR	NR

Author – year		Physical examination	Laboratory findings		Ultrasonographic abnormalities		
		Tenderness	Elevated leukocyte	C-reactive protein levels	Gallstones, sludge	Wall thickening ≥ 3 mm, pericholecystic fluid	Murphy's sign
Abi-Haidar et al 2012	**PTGBD group**	Yes	Yes	Yes	Yes	Yes	Yes
	**LC in control group**	Yes	Yes	Yes	Yes	Yes	Yes
	**OC in control group**	Yes	Yes	Yes	Yes	Yes	Yes
Rodrìguez-Sanjuàn et al 2012	**PTGBD group**	Yes	Yes	Yes	Yes	Yes	Yes
	**LC in control group**	Yes	Yes	Yes	Yes	Yes	Yes
	**OC in control group**	Yes	Yes	Yes	Yes	Yes	Yes

Author – year		Physical examination	Laboratory findings		Ultrasonographic abnormalities		
		Tenderness	Elevated leukocyte	C-reactive protein levels	Gallstones, sludge	Wall thickening ≥ 3 mm, pericholecystic fluid	Murphy's sign
Melloul et al 2011	**PTGBD group**	Yes	Yes	Yes	NR	Yes	NR
	**LC in control group**	Yes	Yes	Yes	NR	Yes	NR
	**OC in control group**	Yes	Yes	Yes	NR	Yes	NR
