# Supplementary figures and images for: Management of Acute Cholecystitis in High-Risk Patients: Percutaneous Gallbladder Drainage as a Definitive Treatment vs. Emergency Cholecystectomy—Systematic Review and Meta-Analysis
Source: J Clin Med. 2023 Jul 26;12(15):4903. doi: 10.3390/jcm12154903 (PMC10419867; doi:10.3390/jcm12154903)

Supplementary Figure S1. Prisma flow chart of literature search

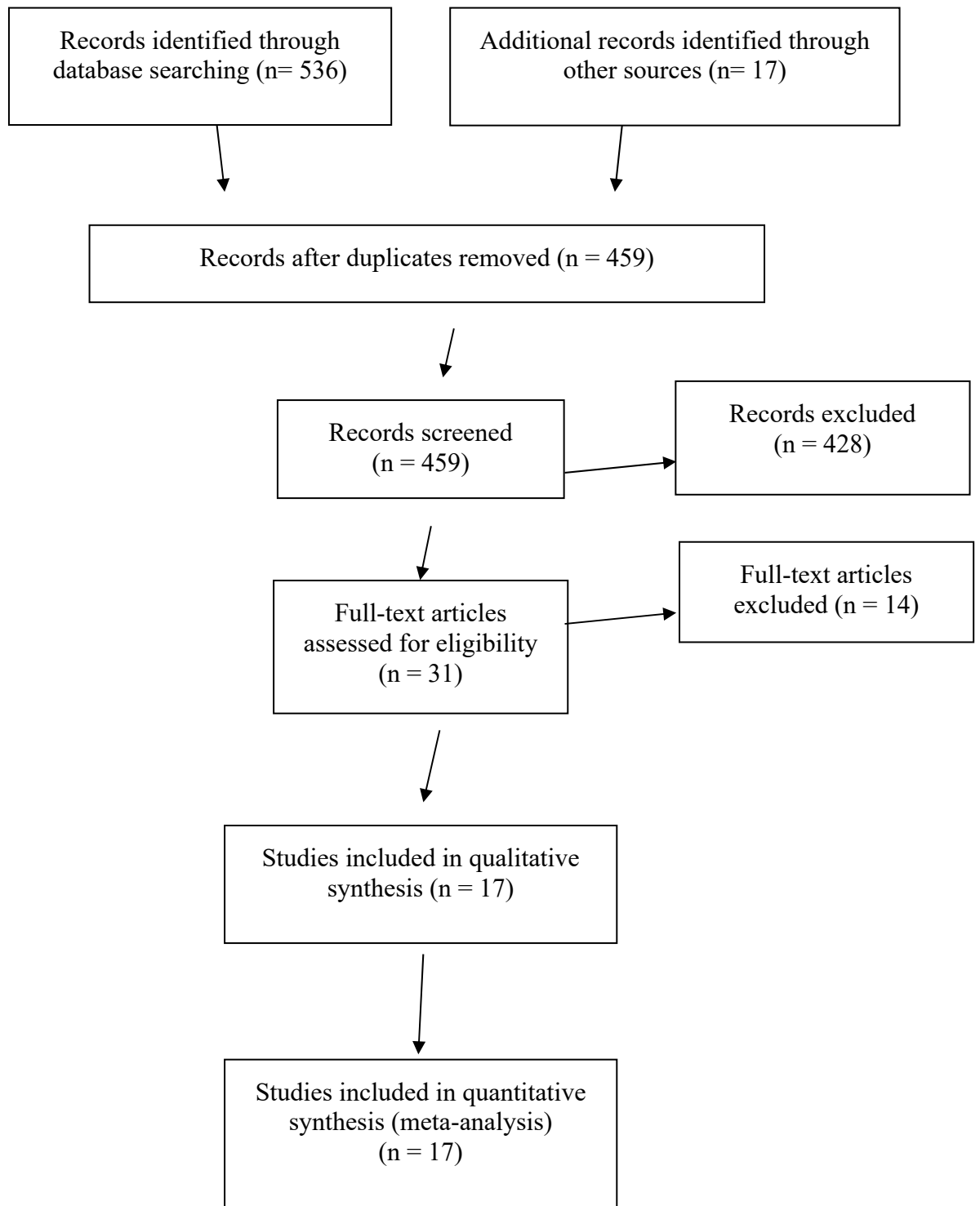

Supplement: Supplementary file 1 [file jcm-12-04903-s001.zip › SDC Fig 1.pdf]

Supplementary Figure S4. Forest plot of abdominal abscess in PTGBD vs EC

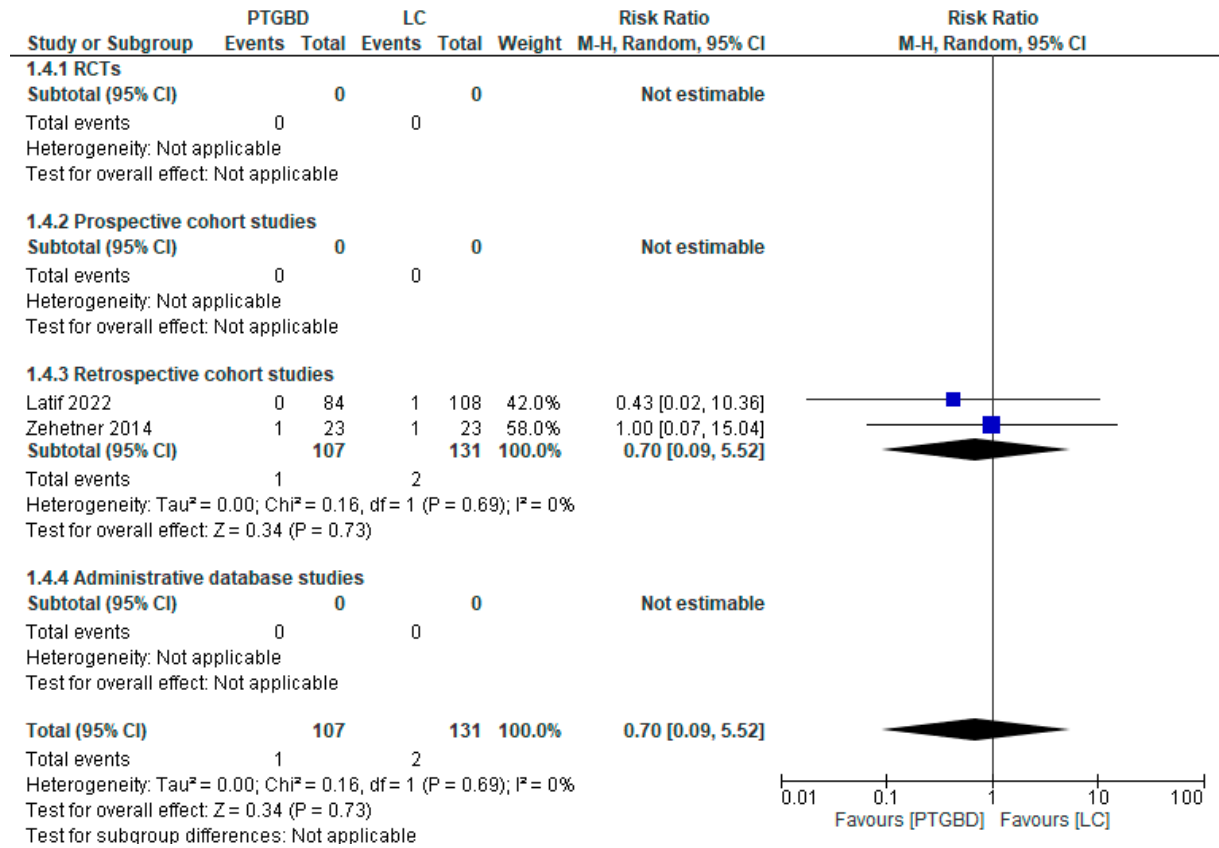

Supplement: Supplementary file 1 [file jcm-12-04903-s001.zip › SDC Fig 4.pdf]

Supplementary Figure S5. Forest plot of major postoperative complications between PTGBD vs EC

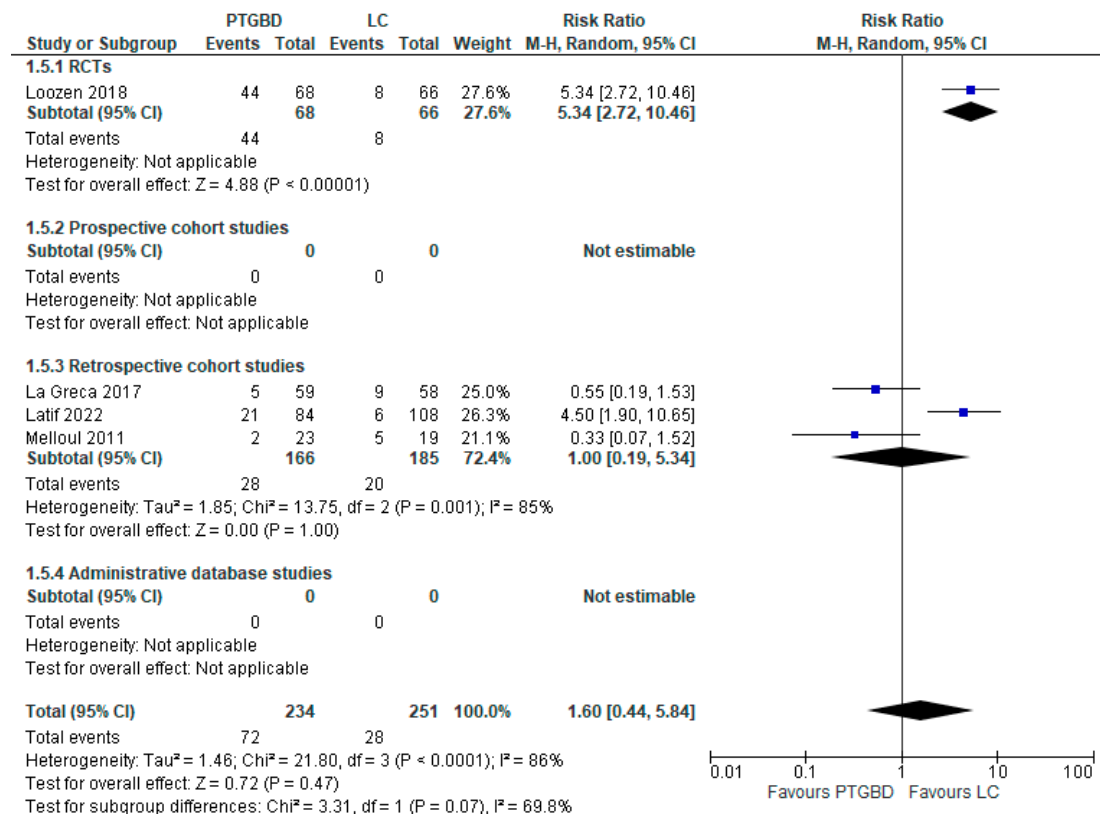

Supplement: Supplementary file 1 [file jcm-12-04903-s001.zip › SDC Fig 5.pdf]

Supplementary Figure S6. Forest plot of length of hospital stay between PTGBD vs EC

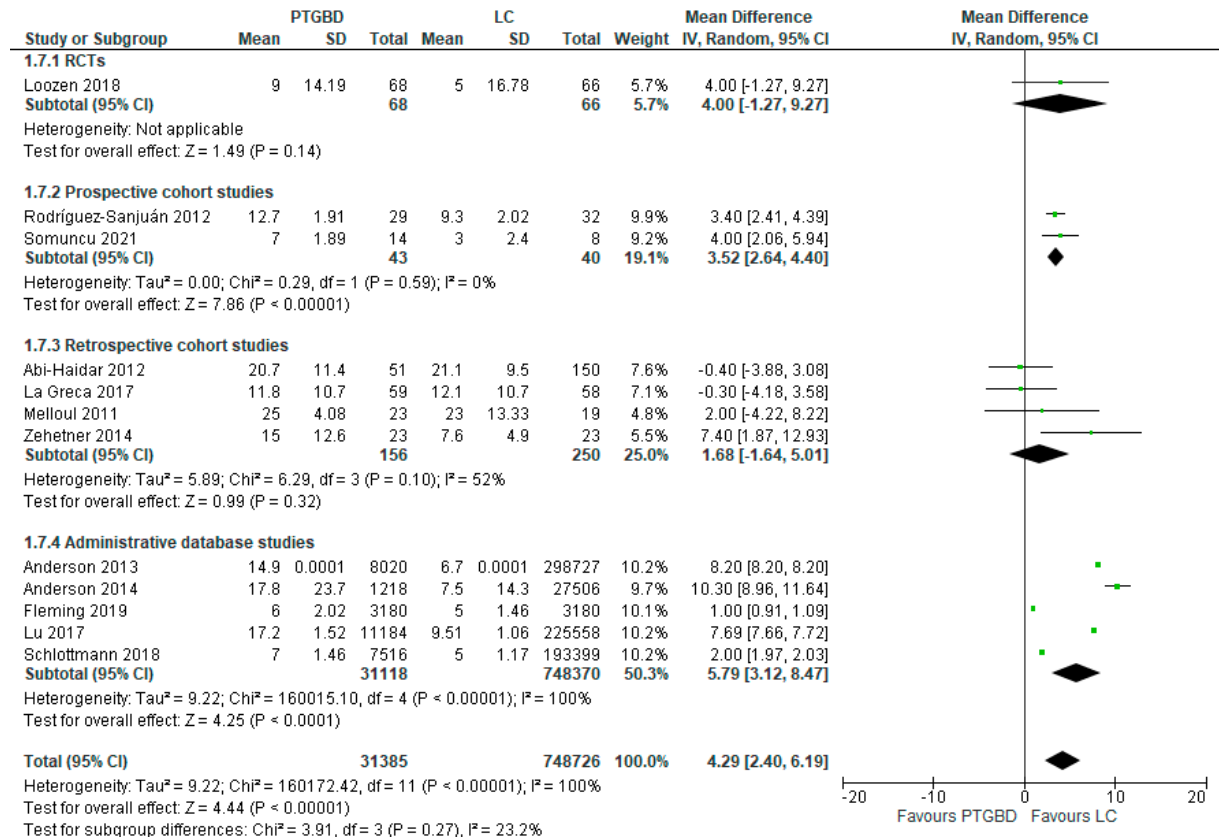

Supplement: Supplementary file 1 [file jcm-12-04903-s001.zip › SDC Fig 6.pdf]
